# HAPPI: an online database of comprehensive human annotated and predicted protein interactions

**DOI:** 10.1186/1471-2164-10-S1-S16

**Published:** 2009-07-07

**Authors:** Jake Yue Chen, SudhaRani Mamidipalli, Tianxiao Huan

**Affiliations:** 1 School of Informatics, Indiana University – Purdue University, Indianapolis, IN, USA; 2 Department of Computer & Information Science, Purdue University, Indianapolis, IN, USA; 3 Indiana Center for Systems Biology and Personalized Medicine, Indianapolis, IN, USA; 4 School of Life Sciences, Shandong University, PR China

## Abstract

**Background:**

Human protein-protein interaction (PPIs) data are the foundation for understanding molecular signalling networks and the functional roles of biomolecules. Several human PPI databases have become available; however, comparisons of these datasets have suggested limited data coverage and poor data quality. Ongoing collection and integration of human PPIs from different sources, both experimentally and computationally, can enable disease-specific network biology modelling in translational bioinformatics studies.

**Results:**

We developed a new web-based resource, the Human Annotated and Predicted Protein Interaction (HAPPI) database, located at . The HAPPI database was created by extracting and integrating publicly available protein interaction databases, including HPRD, BIND, MINT, STRING, and OPHID, using database integration techniques. We designed a unified entity-relationship data model to resolve semantic level differences of diverse concepts involved in PPI data integration. We applied a unified scoring model to give each PPI a measure of its reliability that can place each PPI at one of the five star rank levels from 1 to 5. We assessed the quality of PPIs contained in the new HAPPI database, using evolutionary conserved co-expression pairs called "MetaGene" pairs to measure the extent of MetaGene pair and PPI pair overlaps. While the overall quality of the HAPPI database across all star ranks is comparable to the overall qualities of HPRD or IntNetDB, the subset of the HAPPI database with star ranks between 3 and 5 has a much higher average quality than all other human PPI databases. As of summer 2008, the database contains 142,956 non-redundant, medium to high-confidence level human protein interaction pairs among 10,592 human proteins. The HAPPI database web application also provides …” should be “The HAPPI database web application also provides hyperlinked information of genes, pathways, protein domains, protein structure displays, and sequence feature maps for interactive exploration of PPI data in the database.

**Conclusion:**

HAPPI is by far the most comprehensive public compilation of human protein interaction information. It enables its users to fully explore PPI data with quality measures and annotated information necessary for emerging network biology studies.

## Background

Protein-protein interactions (PPIs) is  an important foundation for understanding how biological processes take place in cells, how cellular signals are modulated, and how molecules orchestrate in response to external environmental stimuli [1]. High-throughput projects that map protein-protein interactions in model organisms were first initiated less than a decade ago, including those for *Saccharomyces cerevisiae*, (resulted in the detection of 957 putative interactions involving 1,004 proteins) [2], *Drosophila melanogaster *(20,405 interactions from 7048 proteins), *Caenorhabditis elegans *(~5,500 interactions), and *Mus musculus *[3-5]. In 2003, Chen *et al. *first reported the generation of 13,656 high-throughput human protein interactions in homogenized human brain using a random yeast two-hybrid platform [6]; in 2005, Stelzl *et al. *identified 3,186 mostly novel interactions among 1,705 human proteins [7]; then, Rual *et al. *reported the mapping of ~2,800 proteins in a human protein-protein interaction network [8]; in 2007, Ewing *et al. *reported a large-scale study of protein-protein interactions in human cells using a mass spectrometry-based approach, producing a data set of 6,463 interactions among 2,235 distinct human proteins [9].

These high-throughput experimental determinations of PPIs have led to an influx of PPI experimental data. By early 2008, BioGrid reported a comprehensive collection of 198,000 protein and genetic interactions from major organisms, including *S. cerevisiae*, *S. pombe*, *D. melanogastor*, *C. elegans*, *M. musculus*, and *H. sapiens *[10]. However, the coverage of data directly captured from experimental platforms in human is still quite poor. In the most recent release 7 of the Human Protein Reference Database (HPRD) [11], there are only 38,167 protein interactions reported – an average of only 1.5 interactions reported for each of the 25,661 human proteins included in HPRD.

While it remains an open question how many measurable human protein interactions there are, the use of PPI data in building disease-relevant molecular interaction network models has already emerged as a major theme for "translational bioinformatics", studies that aim to facilitate the transformation of bioinformatics discoveries from "Omics" experiments into biomedical applications via bi-directional information exchange [12,13]. Recent research studies have shown that, by building comprehensive disease-relevant PPI sub-networks, researchers can generate and validate biological hypothesis that could lead to novel biomarkers or therapeutic developments for many complex diseases such as Huntington's disease, Alzheimer's disease, Breast Cancer, Fanconi Anemia, and Ovarian Cancer [14-18]. These studies, however, were primarily based on available human PPIs in existing PPI database repositories with limited coverage and/or uncertain qualities. It is expected that new comprehensive database collections of human PPIs, with expanded data coverage and quantifiable reliability measures, could significantly enhance the impact of future network modeling research.

Several human PPI databases have begun to expand experimental human PPI data coverage that is bottlenecked by experimental data throughput and cost. There are four common approaches for PPI data expansions: 1) manual curation from the biomedical literature by experts; 2) automated PPI data extraction from biomedical literature with text mining methods; 3) computational inference based on interacting protein domains or co-regulation relationships, often derived from data in model organisms; and 4) data integration from various experimental or computational sources. Partly due to the difficulty of evaluating qualities for PPI data, a majority of widely-used PPI databases, including DIP, BIND, MINT, HPRD, and IntAct [11,19-22], take a "conservative approach" to PPI data expansion by adding only manually curated interactions. Therefore, the coverage of the protein interactome developed using this approach is poor. In the second literature mining approach, computer software replaces database curators to extract protein interaction (or, association) data from large volumes of biomedical literature [23]. Due to the complexity of natural language processing techniques involved, however, this approach often generates large amount of false positive protein "associations" that are not truly biologically significant "interactions". The advantages of computational inferences are attributable to various biological models that can be used to expand data coverage. For example, the HPID database was developed from existing structural and experimental data by homology searching [24]; OPHID was also constructed by mapping interacting proteins from model organisms to their human protein orthologs [25]. In an integrative approach, PPI data from different sources are evaluated and combined, thus providing maximal likelihood for quality and coverage. For example, the STRING database (version 7) [26] has now integrated known and predicted interactions from a variety of sources, and covers all domains of life (prokaryotes to higher eukaryotes). Xia *et al. *applied a probabilistic model and integrated 27 heterogeneous genomic, proteomic and functional annotation datasets to predict human PPI networks [27]. UniHI and IntNetDB are both based on several major interaction maps derived by computational and experimental methods [27,28]. The challenge for the integrative approach is how to balance quality with coverage. In particular, different databases may contain many redundant PPI information derived from the same sources, while the overlaps between independently derived PPI data sets are quite low [29,30].

In this work, we describe a new PPI web database resource, Human Annotated Protein-Protein Interactions (HAPPI), located at . As of early 2008, HAPPI (version 1.1) contains 142,956 non-redundant, medium to high-confidence human protein interaction pairs among 10,592 human proteins identified by UniProt protein names. The HAPPI database aims to become the most comprehensive public compilation of human protein interaction information. The protein interactions are integrated from multiple data sources including both experimental and computationally-derived PPI. Each protein interaction in HAPPI is assigned a PPI confidence grade of 1, 2, 3, 4, or 5 to help users evaluate the reliability and confidence of reported interactions. Each interaction is computationally annotated with information including biological pathways, gene functions, protein families, protein structures, sequence features, and literature sources. These database capabilities will enable both biomedical researchers and network biology users to evaluate the biological significance of specific protein interactions, from which they can build network models for future translational bioinformatics research.

## Methods

**Figure 1:**
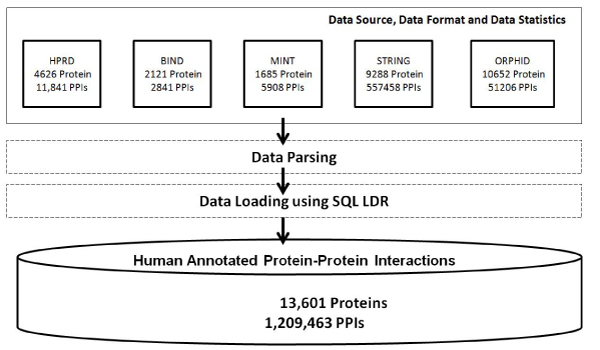
**An overview of the data integration process for developing the HAPPI database**.

Human protein interaction data were collected, extracted, and integrated from the HPRD [11], BIND [20], MINT [21], STRING [26], and OPHID [25] databases, using data warehousing techniques. The primary reason for the choice of these databases was that these sources are relatively complementary to each other and representative of PPIs derived from a variety of methods, including high-throughput experimental PPIs (from HPRD and BIND), literature-curated PPIs (from BIND), text-mined PPIs (from STRING), and computational predicted PPIs (from STRING and OPHID). An overview of the data integration process that involves several of these existing public-domain PPIs databases is shown in Figure 1. The data integration process consists of extracting, transforming, and loading (ETL) of data from downloadable forms of these databases, using PERL and the Oracle 10*g* database server. To take into account of PPIs derived from different data sources, we adopted the data source naming standard from the OPHID database. In particular, for human PPI data from HPRD, BIND, and MINT, we directly used these original database names as the data source names. For data integrated from the STRING database, we used *eSTR* to represent the "experimentally derived subset of STRING interactions", and *pSTR* to represent the "predicted/computationally-derived subset of STRING interactions".

### Data model

**Figure 2:**
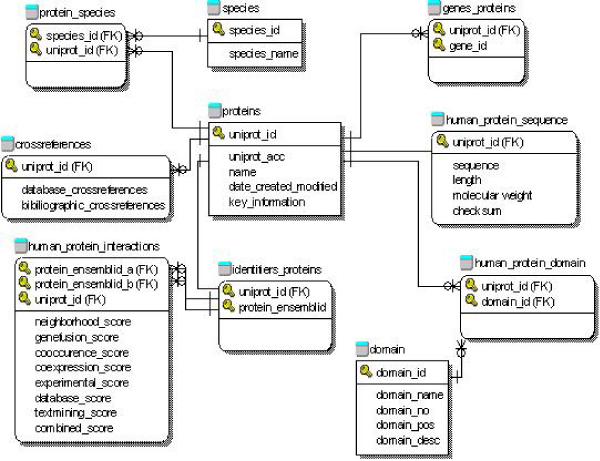
**An entity-relationship data model for the HAPPI database**.

We represented the semantic relationships among different concepts involved in protein interactions as an Entity-Relationship (ER) data model shown in Figure 2, using the Logical Data Structure (LDS) notation as described in [31]. According to this model, each human protein was identified by a unique UniProt ID [32], which could be further linked to other protein/gene identifier systems in other reference databases such as the Ensembl ID from the Ensembl database [33], and comprehensive bioinformatics annotation data stored in other existing biological database resources, such as Pfam [34] that provides information on protein families and domains. Each pair of protein interactions was identified by a pair of protein Uniprot IDs or gene Ensembl IDs to accommodate protein interactions inferred from co-expressed genes from DNA microarrays or co-occurring gene names from text mining, along with several different types of quality scores from the source.

### Interaction ranking model

We developed a unified scoring scheme to assess the reliability of integrated human protein-protein interactions from the public domain. First, an interaction scoring system for each individual data source is either preserved (e.g., adoption of the "combined_score" from STRING) or created (e.g., for OPHID). In the later case, we assigned a heuristic confidence score *S*_*i *_(between 0 and 1) to each interaction pair, based on the type of its experimental/computational derivation method and the database source. *S*_*i *_provided an estimate of the degree of reliability of user confidence in the interaction data. Therefore, the more trustworthy the experimental or computational protocols were, the higher the confidence score (*S*_*i*_) was. Second, to combine the individual confidence scores from different sources into a final *h*_*score *_for the interaction, we used the following formula:



where *N *represented the count of different data sources and conditions, for each of which an independent assessment of protein interaction reliability score, *S*_*i*_, exists. The *h*_*score *_ranges in value between 0 and 1. Third, to convert *h*_*score *_to ranks, we use a ranking method that works in principle by clustering the interactions with closely-related *h*_*score *_values for all interactions managed in the HAPPI database (see supplemental material for details). Then, a five-star ranking model was developed to set the cut-off threshold at the *h*_*score *_distribution cluster boundary. The results are summarized in Table 1. Because the *h*_*score *_values for both high-throughput experimental data (default is 0.75) and curated experimental data from BIND, HPRD, and MINT (default is 0.80) are above 0.75, we therefore selected a combined score of *h*_*score *_>= 0.75, or a final star rank of 4 or 5, as the minimal criteria for reporting interactions and their statistics for HAPPI. A complete initial scoring scheme to assess the reliability of human protein-protein interactions is shown in Additional file 1.

**Table 1:** HAPPI database protein interaction data quality grade and coverage.

**Star Grade**	**Quality Description**	***h-score *range**	**Interaction Count**
1	noisy and uncertain interactions	[0, *0.25*)	546,136
2	low-confidence interactions	[0.25, 0.45)	378,300
3	average-confidence interactions	[0.45, 0.75)	142,071
4	decent-confidence interactions	[0.75, 0.90)	67,462
5	high-confidence interactions	[0.90, 1)	75,494

### Data annotation

All interacting proteins in the HAPPI database were annotated with gene function, pathway, protein domain, protein structure, and sequence feature map data. The data were separately imported into the Oracle 10*g* data warehouse from UniProt [32], GenBank [35], HUGO Nomenclature [36], Ensembl [33], PubMed [37], PDB [38], Pfam [34], and KEGG [39] databases. Altogether, we organized inside the data warehouse 70,829 curated human proteins and their descriptions, of which 13,601 proteins contain protein interaction information in the HAPPI database. We kept 361,975 literature abstract IDs where human gene/protein co-occurrence was detected by the STRING database, 52,186 protein domains/families from Pfam, 715 pathways from KEGG, 2,282 protein 3-D structures from PDB, and 76,797 annotated human gene features from GeneBank. All the information was linked to the original source databases on the HAPPI web site, so that HAPPI users can navigate to database sources to determine the reliability of queried PPIs.

### Quality assessment

In this study, we chose to apply evolutionarily conserved co-expression pairs to the assessment and comparisons of PPI data qualities for different sources, including the HAPPI database. High-quality conserved gene co-expression profiles were used to assess protein interaction quality. Many protein interaction data sets were cross-validated with human gene co-expression profiles such as [40]. While interacting proteins may share highly similar gene expression profiles, it was often suggested that such expected correlation between protein interactions and gene expression is quite weak in human and in *transient *protein interactions. Furthermore, comprehensive expression profiles are difficult to compile for all cellular conditions. To improve the development of a co-expression based confidence measure for interacting proteins, Tirosh and Barkai showed that a method using co-expression of orthologs of interacting partners performed quite well [41]. Their method was based on the assumption that conserved co-expression relationship preserved true protein interactions that required the presence of both interacting proteins through evolution. Therefore, it is more sensitive overall than using information purely from the organism, e.g., simple co-expression, cellular co-localization, and similarity in gene's gene ontology functional annotations. In a similar study, Bhardwaj and Lu also verified that reliable predictions of interactions from heterogeneous data sources could be strengthened by evolutionary conserved gene co-expression measurements [42].

Our computational method was based on the degree of overlap between protein interactions and the use of an evolutionarily conserved co-expressed gene data set called MetaGene. MetaGene consists of 22,163 evolutionary conserved co-expression relationships from humans, flies, worms, and yeast, based on the analysis of over 3182 published DNA microarray experiments by Stuart *et al *[43]. It is a comprehensive compilation of evolutionary conserved gene co-expression pairs from a diverse set of DNA microarray experiments that were obtained from four different organisms: 1,202 DNA microarrays from *H. sapiens*, 979 from *C. elegans*,155 from *D. melanogastor*, and 643 from *S. cerevisiae*. The relative quality of each PPI database, including HAPPI, OPHID [25], IntNetDB [27], ProNet [44], UniHI [28], and HPRD [11], was estimated as the count of overlaps between protein interactions in the PPI database of interest and MetaGene conserved co-expressed gene pairs. The human subset of MetaGene data involves 6,591 human genes and 22,154 MetaGene co-expression gene pairs. 6,297 of the 22,154 human MetaGene co-expression gene pairs can be found in the union (*U*_0 _set) of all the known human PPI databases, including HAPPI, OPHID, IntNetDB, ProNet, UniHI, and HPRD; furthermore, 6,145 of the 6,297 MetaGene pairs form a large connected MetaGene co-expression association network that showed the *scale-free *property commonly observed of most molecular interaction networks. Therefore, we regarded 6,145 Metagene pairs (M0 Set) to be most relevant high-quality subset of U0 and could be used as a gold standard for evaluating unknown PPIs from large databases. To facilitate comparisons of overlaps for different databases with MetaGene, we also developed an artificially synthesized protein-protein "random interaction" set (*R*_0 _Set) of 37,000 PPIs (comparable to the size of all PPIs in HPRD), by randomly reconnecting proteins observed in *U*_0_. Therefore, the lower-bound of any protein interaction data set derived from *U*_0 _could be given by counting the overlap between *R*_0 _and *M*_0_. To adapt to the different sizes of PPI databases, we took a random sample of 1000 PPIs each time from each database in comparison (including *R*_0_), and repeated this random sampling process 1000 times to obtain a distribution of *normalized *overlap counts with *M*_0_.

## Results

HAPPI was developed as a web-based PPIs database application and is freely accessible to the public at . In the current release, HAPPI contains 13,601 proteins and 1,209,463 PPIs integrated from five databases collected with both experimental and computationally methods as described in the previous section. Users of the HAPPI web application software can search for PPIs using common protein identifiers. Typical web query results display all HAPPI PPIs at a default quality grade (star rank 3 and above). Users can drill down to explore annotations of the protein interaction or proteins involved.

### Assessing data quality

While there are several methods for validating PPI data, including those based on interacting domains, gene co-expression profiles, or gene ontology (GO) annotation semantic distances [42,45-49], we assessed the quality of the new HAPPI database by comparing the extent of overlap between PPIs and MetaGene pairs, using a new computational approach described earlier in the Method section.

**Figure 3:**
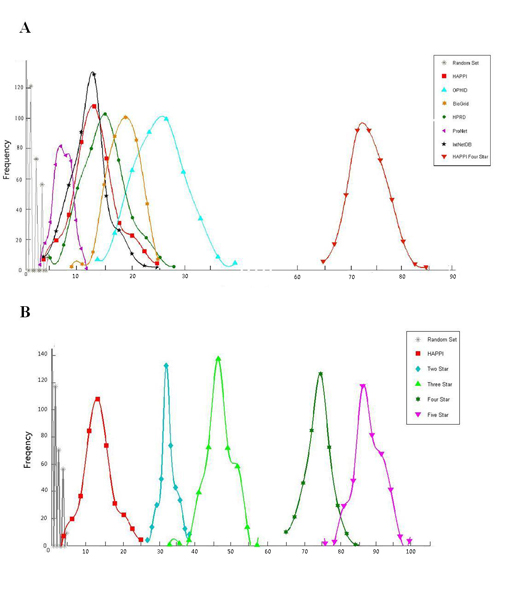
**Degree of overlaps between randomly selected protein interaction pairs in selected protein interaction databases and MetaGene pairs**. We randomly selected 1,000 protein-protein interactions, and counted the numbers of protein interaction pairs overlapped with conserved co-expression pairs in the MetaGene database. This sampling and MetaGene overlapping process was repeated 1000 times for each protein interaction database (including the *random database*). The resulting distributions of overlaps are show as profiles on the graph. **3A**. Comparisons of Metagene overlaps for major human PPI databases, including: HAPPI, OPHID, ProNet, BioGrid, and IntNetDB, and HAPPI 4-star subset. **3B**. Comparisons of Metagene overlap for different quality grade subsets of the HAPPI database, including: HAPPI (all), HAPPI 2-star, HAPPI 3-star, HAPPI 4-star, and HAPPI 5-star subsets. There are 6145 co-expression pairs in MetaGene database in total. And there are 22154 PPIs in HAPPI, HPRD etc. The x-axis represents the number of overlap protein pairs in HAPPI and MetaGene when we random selected 1000 PPIs. The scale of x-axis is calculated as 1/((6145/22154)*1000).

In Figure 3A and 3B, we show the sample count frequency distribution of overlaps between human PPIs from several databases of interest and MetaGene gene pairs. The x-axis represents the count of PPI database and MetaGene overlaps, ranging from 0 to 1000 (total PPIs in each sample is 1000). The y-axis represents the total sample frequency for a specific overlap count value, also ranging from 0 to 1000 but mostly within 200. The cumulative count frequency for each PPI database, including the "Random Set" (see Methods for details), should sum to 1000 (1000 random samplings were performed for each database). As we described in the Methods, we can assess the overall PPI database quality based on the overlap of its PPIs with high-quality MetaGene gene pairs.

Figure 3A shows that the 4-star quality grade HAPPI database subset has the highest MetaGene overlap at approximately 72 out of 1000, among all databases compared (including UniHI, at approximately 8 overlaps, data not shown). The overall quality of the HAPPI database (at all star grades) is comparable to that of the recently published IntNetDB or HPRD (at approximately 13–15 overlaps overall), still better than that of the ProNet [50] database (manually curated data set initially made public as the first database for human protein interactions; at approximately 8 overlaps overall). The overall quality of HAPPI database at all star grades is not as good as the BioGrid (at approximately 19 overlaps) or the OPHID database (at approximately 27 overlaps but with a wide spread), primarily because HAPPI database at one-star quality grade contains many literature mining based co-citation data that do not physically interact. The result also suggests that the overall quality of OPHID database exceeds that of the reference curated HPRD database. We believe that this is primarily due to the challenge in identifying false positive interactions inherent in many experimentally-derived high-throughput PPI data, which HPRD also included with minimal additional validations. The OPHID database incorporated functionally conserved sequence and structure information such as conserved interacting domain pairs (as in the case of OPHID), for developing and filtering human PPI data collected from different organisms, and may have therefore enriched its database with these computationally-derived plausible PPIs.

In Figure 3B, we show a sample frequency distribution of MetaGene overlaps among different quality grades of the HAPPI database subsets. The figure shows that while the overall data quality for the entire HAPPI database of 1.2 million PPIs may be relatively un-impressive (at an average MetaGene overlap of 14 out of 1000 in each sample), the remaining 650,000+ HAPPI database PPIs at star quality grades of 2 and above have an overall quality better than that of any of the existing public databases in the comparison, including the OPHID database. The average count of MetaGene overlaps also improves as the quality grade improves, at approximately 31 for 378,300 2-star PPIs, 47 for 142,071 3-star PPIs, 75 for 67,462 4-star PPIs, and 87 for 75,494 5-star PPIs. While the community knowledge of what constitutes "true protein interactions" in all cellular conditions remain poor, it is still challenging to validate the rest of PPIs that MetaGene data do not cover. However, our results show that the HAPPI database, particularly for star grades of 3, 4, and 5, clearly contains much higher true positive PPI interactions than all other known human PPI databases. For that reason, we only report HAPPI database results with star grades of 3 and above in our database's web user interface.

**Figure 4:**
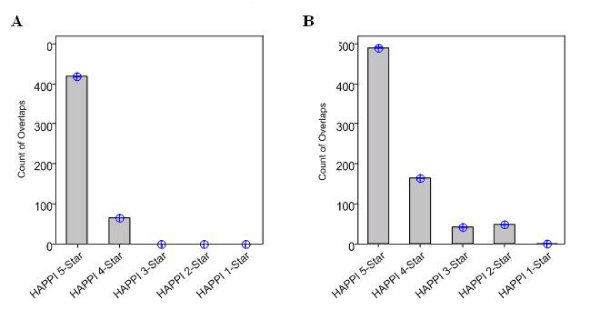
**Count of PPI overlaps between HAPPI database subsets of different quality ratings and the HPRD/OPHID database**. We randomly selected 1,000 PPIs each from HAPPI database 5-, 4-, 3-, 2-, and 1-star subsets and counted each of its overlap with protein interactions with the HPRD database or the OPHID database separately. We repeated this process 100 times for each overlap. The average and 95% confidence interval (CI) on the count of overlaps are shown in the HPRD database (**Panel A**) and the OPHID database (**Panel B**).

We also analyzed PPI overlaps between HAPPI database subsets of different quality grades and two reference PPI databases. In Figure 4A, we show that an average of approximately 410 out of 1,000 (41%) randomly selected HAPPI 5-star PPIs overlap with the HPRD database. This high-degree of overlap drops to approximately 8% for HAPPI 4-star PPIs, and almost nothing for HAPPI 3-star, 2-star, and 1-star subsets. In Figure 4B, we show that an average of nearly 500 out of 1,000 (50%) randomly selected HAPPI 5-star PPIs can be overlapped with the OPHID database. This high-degree of overlap drops to approximately 17% for HAPPI 4-star PPIs, 4% for HAPPI 3-star PPIs, 5% for HAPPI 2-star PPI, and eventually to nothing to HAPPI 1-star subsets. Recall that Fig 3 suggested that OPHID has a slightly higher overall PPI data quality level than HPRD, and that HAPPI 4-star or HAPPI 5-star subsets are two of the best PPI data sources compared. It is therefore not surprising that OPHID and HAPPI 4-star or 5-star subsets are more consistent with each other. The low degree of overlaps with the reference databases at lower HAPPI quality grades are expected, because of the much higher coverage of PPIs and lower confidence in data quality in HAPPI 3-star, 2-star, and 1-star subsets.

### Querying the database

HAPPI enables users to retrieve human PPI data through multiple types of protein identifiers, such as UniProt IDs, Swiss-Prot accession numbers, RefSeq IDs, or IPI accession numbers, at its query home page. Query results that contain protein interaction data and quality rank are shown in a single web page as a data table. The query result is available for download either in a Molecular Interaction (MI) format recommended by the Proteomics Standard Initiatives (PSI) or in a Graph Markup Language (GML) format recommended by the International Molecular Exchange Consortium. Additional annotation details of the protein or protein interaction can be queried and retrieved online by selecting the hyperlinks in the protein interaction result page.

### Viewing and exploration of results

**Figure 5:**
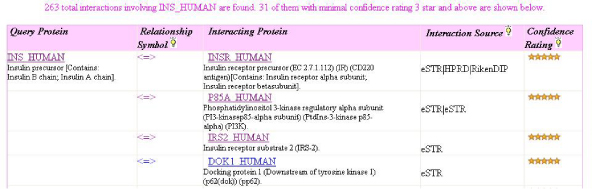
**The user interface (partial) that lists protein interactions retrieved by searching HAPPI with the query protein INS_HUMAN (insulin precursor protein)**. Both interactions shown here are derived from multiple data sources and have 5-star confidence ratings.

HAPPI users can retrieve a list of protein interactions showing the following fields in a table: the query protein, a relationship symbol (currently implemented as bi-directional binding, represented as "<=>"), the data source of the interaction, and a confidence rating of 1 to 5 stars. Figure 5 shows an example (in a partial view) of protein interaction results retrieved with the query INS_HUMAN, insulin precursor protein. Note that we relaxed the interaction criteria here to allow the display of every interaction having a 3-star or higher confidence score rating. Second, the user can navigate to the protein information page to learn about additional annotation details of the interacting protein, and to link out to a wide variety of protein annotation databases. Third, the user can also navigate to the protein interaction pair details page to further examine biological relationship evidence that may exist between interacting proteins. For example, knowing previously that INS_HUMAN interacts with INSR_HUMAN (insulin receptor precursor protein) with high confidence (at the 5-star level), users can learn from these protein descriptions that it is the processed forms, not the precursor forms, of both insulin and insulin receptor dimers, that actually bind to each other. In addition, the user can learn that this interaction is involved in several biological processes together, because the interacting proteins have several pathways such as insulin signalling, type II diabetes, and DLPRA in common. Various other types of annotation information are also available for the interacting proteins within the same web page for users to take advantage of. These types of annotation information are: top gene/protein in literature co-occurrence references, which may help users find evidence for protein interactions; protein family/domain annotation, which may help users to identify interacting domains; side-by-side display of the 3-D structures of interacting proteins, which may help structural biologists recognize matching protein domains or surfaces for interactions; and head-to-head gene sequence feature alignment, which may help users hypothesize whether a plausible interaction is supported by sequence and its features.

**Figure 6:**
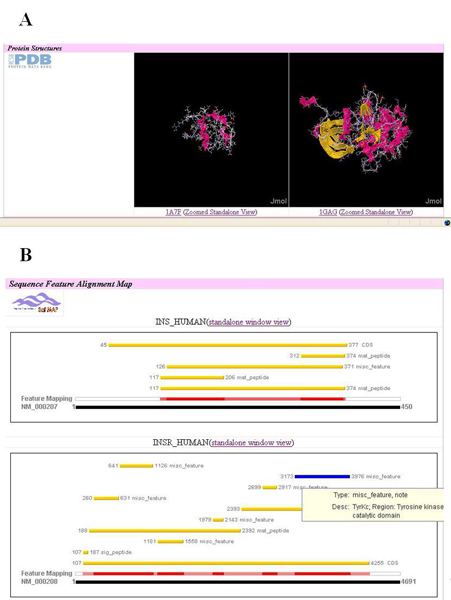
**A partial view of annotated protein interaction features in the HAPPI database**. A) The PDB structure of Insulin precursor protein (1A7F, on the left) is displayed in HAPPI side-by-side with the structure of Insulin receptor precursor protein (1GAG, on the right, in monomer form). B) The gene feature alignments for both the Insulin precursor protein (INS_HUMAN) and the Insulin receptor precursor protein (INSR_HUMAN) are created in real time in HAPPI. A tooltip that labels the top right highlighted sequence feature of Tyr Kinase domain of INSR_Human is also shown.

We created two interactive components in the protein interaction details page: one to explore interacting protein 3D structures and the other to explore interaction protein feature alignments. In Figure 6A and 6B, we show an example of these two components. Two protein PDB structures, one for INS_HUMAN and the other for INSR_HUMAN, are displayed side by side using two JMOL [51] Java Applet Plugins of the web browser (Figure 6A). Once the applet control is activated by a mouse click, the user can adjust the structure viewer's properties for the two proteins side-by-side. Similarly, the user can use mouse-over actions to browse tooltips associated with each sequence feature aligned on top of each of the two protein-coding genes in the Safmap Java Applet viewer (Figure 6B). After extensive interaction with these dynamic components of the HAPPI application, the user may recognize the INSR_HUMAN N-terminus as a signal peptide (confirmed on the SafMap) forming an α-helix sticking out from the Cys-rich ligand binding domain of the insulin receptor. The Tyr kinase domain of the insulin receptor is, however, tucked right on the same side beneath the ligand binding domain of the receptor but away from the α-helix rich body of the molecule. With this exploration under way, it is not difficult to confirm that the INSR dimerization creates a good binding pocket for the small INS peptide, which upon binding further activates the nearby Tyr kinase autophosphorylation, therefore triggering a cascade of signalling events in cells [52].

## Conclusion

HAPPI is by far the most comprehensive public compilation of human protein interaction data that come with a unified framework of interaction data reliability scores. In its current release, the HAPPI database contains 13,601 proteins and 1,209,463 PPIs integrated from several databases derived either experimentally or computationally. By comparing the degree of overlap between PPIs of varying quality grades and evolutionarily conserved co-expressed gene pairs, we assessed the quality of HAPPI. While the overall quality of HAPPI is comparable to that of the HPRD database, HAPPI PPIs with 3-5 star rank levels have a higher average quality than all other human PPI databases considered in this study, which include ProNet, UniHI, IntNetDB, OPHID, HPRD, and BioGrid.

For future HAPPI database releases, we have three plans. First, we wish to continue integrating and linking valuable annotation data into the HAPPI database. Protein interaction data from high-precision text mining projects could be used to improve the validation of high-quality protein interactions as "re-discovered" compared to the findings reported in past literature. Gene co-expression and Gene Ontology data are also candidates for data import next, since they both can help define common functional context in which protein interactions may take place. Second, we plan on applying database customization techniques to improve the user querying experience with HAPPI. For example, we will add control buttons for users to customize interaction data quality filter thresholds, and to select a subset of retrieved protein interactions for downloading into spreadsheet programs. Third, we wish to improve existing PPI data investigation features. For example, we hope to run molecular docking programs and show computationally predicted protein binding constants and binding sites between two proteins. We also plan to improve the interplay between JMOL and Safmap Java Applets so that a highlight of sequence segments in one program may also be highlighted in the other program. With these improvements, we expect the database to play essential roles for biomedical researchers to retrieve trustworthy information on plausible human protein interaction data and for bioinformatics scientists to conduct network biology modeling studies.

## Competing interests

The authors declare that they have no competing interests.

## Authors' contributions

JYC conceived the initial idea, designed the method for the database construction, and drafted the manuscript. SM implemented the design, developed the database from integrated data sets, and implemented the web-based database interface. TH performed database comparisons and evaluations of the database. All authors are involved in the revisions of the manuscript.

## Supplementary Material

Additional file 1 A unified scoring model to assess the reliability of human protein-protein interactions integrated from public protein interaction databases. Click here for file
