# Supplementary material for: HAPPI: an online database of comprehensive human annotated and predicted protein interactions
Source: BMC Genomics. 2009 Jul 7;10(Suppl 1):S16. doi: 10.1186/1471-2164-10-S1-S16 (PMC2709259; doi:10.1186/1471-2164-10-S1-S16)
Supplement: Additional file 1 — A unified scoring model to assess the reliability of human protein-protein interactions integrated from public protein interaction databases. [file 1471-2164-10-S1-S16-S1.docx]

### Additional file 1 – A unified scoring model to assess the reliability of human protein-protein interactions integrated from public protein interaction databases.

| 0.80 | *Curated Human Protein Interactions found in HPRD, BIND,* and MINT |
| --- | --- |
| 0.75 | High-throughput human protein interaction experimental data |
| 0.70 | Human protein interactions in OPHID predicted from mouse and rats |
| 0.65 | High-quality human protein interactions in OPHID predicted from drosophila |
| 0.60 | Medium-quality human protein interactions in OPHID predicted from various mouse, rat, and drosophila projects |
| 0.50 | Human protein interaction data inferred from medium-to-high quality worm and yeast data; high-quality text mining results from STRING |
| 0.40 | Human protein interaction data inferred from low-quality worm or curated/high-quality yeast data (including those from MIPS yeast); medium-quality text mining results imported primarily from the STRING database |
| 0.05-0.35 | Human protein interaction data inferred from non-interaction data sources (indirect association evidence); low-to-medium-quality text mining results imported primarily from STRING database |
